# PAIN PERCEPTION AND PAIN COPING MECHANISMS IN CHILDREN AND ADOLESCENTS WITH JUVENILE FIBROMYALGIA AND POLYARTICULAR JUVENILE IDIOPATHIC ARTHRITIS

**DOI:** 10.1590/1984-0462/;2019;37;1;00006

**Published:** 2018-06-11

**Authors:** Melissa Mariti Fraga, Maria Teresa Terreri, Rafael Teixeira Azevedo, Maria Odete Esteves Hilário, Claudio Arnaldo Len

**Affiliations:** a Universidade Federal de São Paulo, São Paulo, SP, Brasil.

**Keywords:** Musculoskeletal pain, Juvenile arthritis, Fibromyalgia, Pain perception, Psychological adaptation, Dor musculoesquelética, Artrite juvenil, Fibromialgia, Percepção da dor, Adaptação psicológica

## Abstract

**Objective:**

To measure and compare musculoskeletal pain in patients with juvenile fibromyalgia (JFM) and polyarticular juvenile idiopathic arthritis (JIA), and to evaluate and compare pain perception and pain coping mechanisms in these patients.

**Methods:**

In this cross sectional study, we evaluated 150 children and adolescents, and their respective parents, from 3 different groups: JFM, polyarticular JIA, and healthy controls. Pain intensity and pain coping mechanisms were measured using specific questionnaires. Pain perception was evaluated according to three illustrations simulating situations that might cause pain: a shot, a bicycle fall, and social isolation. The patients’ parents also filled out the questionnaires and provided a pain score that matched their child’s perception of pain for each illustration.

**Results:**

The highest pain scores, the lowest pain coping strategy scores, the highest pain perception scores for all three illustrations, and the worse health related to quality of life indicators were observed in the JFM group, when compared to the JIA and control groups. The same pattern was observed with their parents.

**Conclusions:**

Patients with JIA and JFM behave differently in relation to pain perception and the development pain coping mechanisms. Pain should be evaluated from different perspectives for an individualized and efficient treatment of patients.

## RESUMO

**Objetivo:**

Mensurar e comparar a dor musculoesquelética em pacientes com fibromialgia juvenil (FMJ) e em pacientes com artrite idiopática juvenil poliarticular (AIJ); e avaliar e comparar a percepção e o enfrentamento da dor.

**Métodos:**

Foram avaliados, em estudo transversal, 150 crianças e adolescentes (e seus respectivos pais), divididos em três grupos: FMJ, AIJ e controles saudáveis. A mensuração e o enfrentamento da dor foram realizados por meio de instrumentos específicos. Para a avaliação da percepção da dor, desenvolveram-se três vinhetas com simulação de situações que pudessem gerar dor: aplicação de injeção, queda de bicicleta e isolamento social. Os pais e os pacientes responderam individualmente quanto à percepção da dor em cada situação.

**Resultados:**

As maiores notas de dor, os menores escores de enfrentamento da dor, as maiores notas para a percepção da dor nas vinhetas e os piores índices de qualidade de vida relacionada à saúde foram observados nos pacientes com FMJ, quando comparados aos pacientes com AIJ e aos controles. O mesmo padrão foi observado com os respectivos pais.

**Conclusões:**

Pacientes com AIJ e FMJ se comportam diferentemente em relação à percepção da dor e ao desenvolvimento de técnicas para o enfrentamento da dor. A dor deve ser avaliada sob diferentes perspectivas para um planejamento mais individualizado e efetivo do tratamento desses pacientes.

## INTRODUCTION

Musculoskeletal pain is one of the most common complaints from the pediatric age group. Many patients have idiopathic musculoskeletal pain (IMP), which does not necessarily have an apparent cause, is intermittent, and can be disabling, for a minimum period of three months.^1^

Some case series show that 25-40% of children and adolescents with chronic musculoskeletal pain syndrome meet the criteria for juvenile fibromyalgia (JFM).^2^ ^,^ ^3^ Furthermore, the frequency of JFM in schoolchildren is 1.2 to 6.2%.^4^ ^,^ ^5^ It is more common in girls, which indicates a significant prevalence of this disease. It also has an impact on daily activities, which results in a decrease in the health-related quality of life (HRQOL) of patients and their families.

Juvenile idiopathic arthritis (JIA) is a chronic inflammatory disease characterized by arthritis that lasts at least six weeks in at least one joint. Pain is common in patients with JIA, especially in those with polyarticular impairment (five or more inflamed joints). Previous studies have shown that 86% of children with JIA present mild to moderate pain^6^ and that pain and JIA have an impact on HRQOL.^7^ ^,^ ^8^ ^,^ ^9^

Pain, regardless of its cause, is perceived, tolerated and dealt with differently by patients. However, these aspects are rarely studied when JFM and JIA are analyzed.^10^ ^,^ ^11^ ^,^ ^12^ Our pioneering study aimed to measure the pain of patients with JFM and JIA, as well as to quantify their perception of pain, evaluate the way they adapt to the pain and their coping mechanisms.

## METHOD

The present cross-sectional study was carried out in a specialized outpatient clinic of a tertiary hospital. One hundred patients aged 8 to 18 years old were included consecutively: 50 patients with JFM and 50 patients with polyarticular JIA. Patients with JFM met the criteria for fibromyalgia as established by the American College of Rheumatology in 1990.^13^ Patients with polyarticular JIA met the diagnostic criteria of the International League Against Rheumatism.^7^ All patients had a minimum follow-up period of six months.

The control group consisted of 50 randomly selected children and adolescents, chosen in order of when they joined a leisure club. This group was paired off by gender and age, and they had no history of inflammatory disease or previous chronic musculoskeletal pain.

Data was collected with regard to the age of onset, intensity, duration of the pain, and previous painful experiences that were related or unrelated to the disease (surgeries, hospital admissions, laboratory exams / venous punctures, fractures, immobilizations, traumas) and use of analgesic and/or anti-inflammatory medication. For JIA patients, the disease activity and the number of active joints at the time of the interview were evaluated. We used the numerical visual analogue scale (VAS)^14^ to evaluate pain intensity, which was scored from 0 to 10.

Patients reported the intensity of their pain at the time of the interview and the average pain they felt during the previous week. The parents indicated the pain score that they believed their children felt on the day of the interview.

To evaluate pain perception, we developed three vignettes related to situations associated with physical or psychological pain:

1. physical pain related to a traumatic experience (bicycle fall);
2. pain related to a medical procedure (shot); and
3. emotional pain, due to social deprivation (excluded from a group of children).

**Figure 1:**
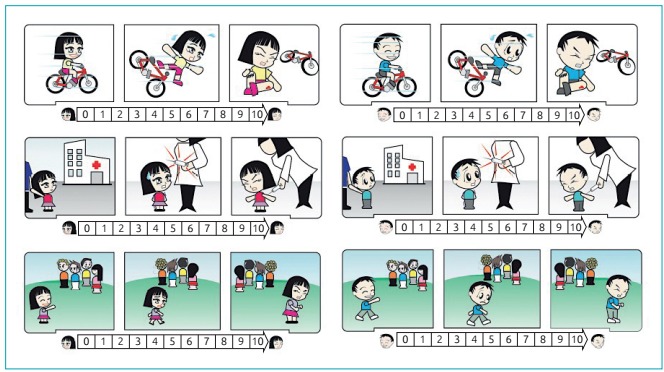
Vignettes of simulated pain situations: trauma after a bicycle crash (first line); administration of a parenteral medication: a shot (second line) and social deprivation caused by a group of children (third line).

A designer drew up the vignettes after a consensus meeting among the researchers (Figure 1). The characters of the vignettes were female and male, and the patients and controls were instructed to assign a score of 0 to 10 (a line 10 cm long) according to the individual’s perception of pain, after looking at each vignette. Parents also gave a score of 0 to 10 for each vignette, assessing the pain their child might experience in each situation. The vignette was presented individually to each participant, one at a time, and in the same order.

To assess pain coping mechanisms, the Waldron/Varni Pediatric Pain Coping Inventory (PPCI) questionnaire, translated into Portuguese, was used.^15^ This tool encompasses the following aspects: cognitive self-instruction (“tell myself to be brave”; “imagine I can make the pain or hurt disappear by myself”; “pretend not to feel pain or have an injury”; “tell myself that it will be all right” “know that I can do something to make the pain or hurt feel better”; “know that I can ask for something to improve the pain or hurt”; “pretend that the pain or hurt doesn’t hurt as much as it really does”), problem-solving (“go to bed”; “ask for medicine”; “ask to go to the doctor”; “ask someone to explain why I hurt”; “put ice or heat on the sore spots”; “go to sleep until it feels better”), distraction (“think about going away on vacation or a trip”; “watch TV”, “play a game”; “eat or drink something”; “try not to think about the pain or hurt”; “ignore the pain or hurt”; “think about happy things”; “play with my pet”; “read a book or color in a coloring book”; “talk about what I did today”), seeks social support (“ask for a hug or a kiss”, “ask someone to understand how much I hurts”, “play with my friends”, “ask my mother, father or friend to sit with me,” “tell my mother or my father,” “ask to stay by myself, “; “squeeze someone’s hand or something else,”; “ask someone to tell me that the pain or hurt will go away and I will feel better”), and catastrophizing/helplessness (“cry or yell”; “think it will just get worse”; “wish for it to go away”; “try to be brave and say nothing”; “get mad or be mean to other people”; “think that I can’t do anything to stop the pain”).

Patients with JIA were evaluated regarding the clinical activity of their disease according to the criteria from Wallace et al.^16^

Patients, controls and their respective parents signed consent forms. The study was approved by the Research Ethics Committee of the Universidade Federal de São Paulo (CEP-UNIFESP 0309/10).

Initially, the data were analyzed descriptively. For the categorical variables, absolute and relative frequencies were calculated. For the numerical variables, summarized measurements were calculated (mean, minimum, maximum, quartiles and standard deviation). The means of the two groups were compared via Student’s t-test for independent samples. To perform an analysis for more than two groups, the means were compared using a variance analysis (ANOVA), followed by multiple comparisons (Duncan’s comparisons for groups with equal variances and Dunnett’s C for groups with different variances - if there was a difference between the means) in order to pinpoint the differences. To verify the normality of the data, the Kolmogorov Smirnov test was used. In the case that there was no normality, the nonparametric Kruskal Wallis test was used, followed by the Mann Whitney test (if there was evidence of different means) with a Bonferroni correction. For the comparison of paired sample means, Student’s t-test was used. To verify associations between the categorical variables, the chi-square or Fisher’s exact tests were used if the samples were small. Linear associations between numerical variables (scales) were analyzed using Spearman’s correlation. The internal consistency of the items that make up each of the five PPCI aspects was evaluated using Cronbach’s alpha. A significance level of 5% was used for all statistical tests.

## RESULTS

Three hundred interviews were performed with 150 patients and their parents. The clinical and demographic data of the patients and the demographic data of the controls are shown in Table 1. We observed that all three groups were homogeneous with regard to the patient’s economic situation, gender, and age at the time of the interview.

Among the 50 patients with polyarticular JIA evaluated (seven had positive rheumatoid factor), the disease was active in 29 (58%) of them at the moment of the evaluation.

**Table 1:** Demographic and clinical data of patients with juvenile fibromyalgia, juvenile idiopathic arthritis and of healthy controls. Patients and controls’ pain scores according to the perspective of the patients and the parents.

	Fibromyalgia (n=50)	Juvenile idiopathic arthritis (n=50)	Health controls (n=50)	p-value
Gender (feminine/masculine)	43/7	41/9	42/8	0.86
Age at the onset of pain (years)*	8.7±3.7	5.4±3.3	-	<0.0001
Length of pain (years)*	4.6 ± 1.9	6.9± 2.3	-	<0.0001
Age of the patient at the time of the interview (years)*Θ	13.5±2.7	12.4±3.5	12.8±2.3	0.13
Pain score at the time of the interview from the patients and controls’ perspective (0 to 10)*	5.9±2.6	2.1±2.4	0.1±0.3	<0.0001
Pain score of the previous week from the patients and controls’ perspective (0 to 10)*	7.8±1.8	3.2±2.6	0.2±0.5	<0.0001
Pain score at the time of the interview from the parents’ perspective (0 to 10)*	5.9±2.9	3.0±2.8	0	<0.0001

*Mean± standard deviation; ΘANOVA with Brown-Forsythe correction (F_2_,_132_=2.04; p-value=0.1337), Kolmogorov-Smirnov test (p-value=0.4272).

### Patients and controls’ pain scores

Twenty-six patients with JFM (52%) and 11 patients with polyarticular JIA (22%) had severe pain (pain score > 8) in the week preceding the interview (p-value <0.0001). Nine of the 50 patients in the JFM group (18%) had used painkillers in the week prior to the interview, while eight of the 50 patients with polyarticular JIA (16%) were on pain medication at the time of the interview. In the group of patients with JIA, we did not observe a statistical difference between the pain score on the day of the interview and the score from the week prior to the interview between the patients who were actively suffering from the disease (arthritis) and those who were not.

There was a difference between the mean age of onset of pain in children in the JFM and JIA groups. The onset of pain began in JIA patients at a younger age (5.4 ± 3.3 versus 8.7 ± 3.7; p <0.0001) and their pain lasted longer (6.9 ± 2.3 versus 4.6 ± 1.9, p <0.0001) (Table 1).

We calculated the intraclass correlation coefficient between the pain scores provided by the children and adolescents of the JFM group and their parents. The value found was 0.716, with a 95% confidence interval (95% CI) ranging from 0.550 to 0.828. In patients with JIA, we found a value of 0.658, with the 95% CI ranging from 0.468 to 0.790.

### Patients and controls’ pain perception

Table 2 shows the pain perception scores in each group interviewed. The scores were obtained from the patients and their respective parents after seeing the vignettes. The group of patients with JFM had the highest average and median pain scores in the three simulated situations presented. Comparing the JFM group scores with those from the JIA group and those from the control group, we found, respectively: 7.5 versus 4.7 versus 4.0 and p <0.0001 for physical pain; 8.5 versus 3.5 versus 7.1 and p <0.0001 for pain related to a medical procedure; and 10.0 versus 6.0 versus 7.0 and p <0.0001 for social deprivation. In the analysis of the vignettes showing a medical procedure and social deprivation, distinct scores were found in the three groups (p <0.0001), with the highest scores occurring in the JFM group, followed by the control group. The parents’ perceptions of pain scores were also higher in the JFM group for the three simulated situations presented.

**Table 2:** Pain perception: mean and median pain score comparisons given by each group (juvenile fibromyalgia, juvenile idiopathic arthritis and the healthy control) after the presentation of the following vignettes: physical trauma (bicycle fall), medical procedure (a shot), and social deprivation (isolation by a group of children).

		Mean	SD	Median	Minimum	Maximum	p-value
Patients and controls							
Physical trauma	JFM	7.5^(1)^	1.8	-	-	-	<0.0001*
	JIA	4.7^(2)^	2.6	-	-	-	
	Control	4.0^(2)^	2.3	-	-	-	
Medical procedure	JFM	8.5^(1)^	2.1	-	-	-	<0.0001*
	JIA	3.5^(3)^	3.2	-	-	-	
	Control	7.1^(2)^	2.2	-	-	-	
Social deprivation	JFM	-	-	10.0^(A)^	4	10	<0.0001**
	JIA	-	-	6.0^(B)^	1	10	
	Control	-	-	7.0^(B)^	0	10	
Parents							
Physical trauma	JFM	6.8^(1)^	2.2	-	-	-	<0.0001*
	JIA	5.4^(2)^	2.9	-	-	-	
	Control	3,7^(3)^	2.6	-	-	-	
Medical procedure	JFM	-	2.6	-	-	-	<0.0001*
	JIA	-	3.3	-	-	-	
	Control	-	2.9	-	-	-	
Social deprivation	JFM	-	-	10.0^(A)^	5	10	<0.0001**
	JIA	-	-	6.5^(B)^	0	10	
	Control	-	-	7.0^(C)^	0	10	

SD: standard deviation; JFM: juvenile fibromyalgia; JIA: juvenile idiopathic arthritis; ^(1), (2) and (3)^ show different means according to the C and Dennet multiple comparisons with a global significance of 5%; ^(A), (B) and (C)^ had different means and a global significance of 5% via the Mann-Whitney tests with the Bonferroni correction; *descriptive level from the ANOVA test; **descriptive level from the Kruskal-Wallis test.

When analyzing each pain situation presented in the vignettes separately, the control group obtained higher scores than the patients with JIA in the social deprivation situation (7.7 versus 6.0, p <0.001) and the pain related to a medical procedure situation (7.1 versus 3.5, p <0.001).

The Spearman correlation coefficient between the pain scores given by parents and by children after seeing the vignettes is presented in Table 3. We observed a statistically significant correlation between the scores of patients with JIA and their parents’ scores, which was not found in the JFM group. In the JFM group, the correlation was low, showing that parents do not have the same perception of pain when compared to their children.

**Table 3:** Pain perception: Spearman’s correlation coefficient between pain values attributed by parents and children for each of the three vignettes (physical trauma/bicycle fall, medical procedure/shot and social deprivation) and between vignettes within the groups of children and adolescents with juvenile fibromyalgia, juvenile idiopathic arthritis and the control group.

Correlation of the parents’ pain score versus the children’s pain score	Fibromyalgia	Juvenile idiopathic arthritis	Control
Physical trauma	0.256	0.467*	0,672*
Medical Procedure	0.244	0.543*	0,675*
Social Deprivation	0.200	0.468*	0,596*

*p-value<0.01.

### Patients’ coping mechanisms

Table 4 shows the summary and mean comparisons of the following items: cognitive self-instruction, problem-solving, distraction, seeks social support, and catastrophizing/helplessness from the PPCI questionnaire for each group of patients and the control group. It was observed that the cognitive self-instruction and distraction means were similar between the JFM and JIA groups (7.6 versus 6.4), and both were higher than those of the control group (3.8). It was observed in the PPCI questionnaire that the patients in the JIA group had higher scores for the following items: problem-solving, seeking social support and catastrophizing/helplessness. The control group had the lowest averages.

**Table 4:** Summary of the measurements and the means of the following items: cognitive self-instruction, problem-solving, distraction, seeks social support, catastrophizing/helplessness from the Pediatric Pain Coping Inventory within the groups of children and adolescents with juvenile fibromyalgia, juvenile idiopathic arthritis and the control group.

	Mean	SD	Minimum	Maximum	Median	p-value*
Cognitive self-instruction (0 to 14)						
Juvenile fibromyalgia	7.6^(1)^	3.7	0.0	14.0	8.0	<0.0001
Juvenile idiopathic arthritis	6.4^(1)^	2.7	1.0	13.0	7.0	
Control	3.8^(2)^	4.2	0.0	14.0	2.0	
Problem-solving (0 to 20)						
Juvenile fibromyalgia	7.8^(B)^	3.9	0.0	15.0	8.5	<0.0001
Juvenile idiopathic arthritis	11.1^(A)^	3.7	4.0	19.0	11.0	
Control	5.8^(C)^	3.2	0.0	15.0	6.0	
Distraction (0 to 18)						
Juvenile fibromyalgia	6.5^(A)^	4.1	0.0	18.0	6.5	<0.0001
Juvenile idiopathic arthritis	7.6^(A)^	4.0	0.0	17.0	9.0	
Control	3.4^(B)^	3.1	0.0	11.0	3.0	
Seeks social support (0 to 18)						
Juvenile fibromyalgia	6.4^(B)^	3.8	0.0	18.0	6.0	<0.0001
Juvenile idiopathic arthritis	8.8^(A)^	4.3	0.0	18.0	9.0	
Control	4.4^(C)^	3.2	0.0	11.0	4.0	
Catastrophizing/Helplessness (0 to 12)						
Juvenile fibromyalgia	5.0^(2)^	2.3	1.0	11.0	5.0	<0.0001
Juvenile idiopathic arthritis	6.5^(1)^	2.5	1.0	12.0	6.0	
Control	3.2^(3)^	1.5	0.0	7.0	3.0	

SD: standard deviation; ^(A), (B) and (C)^ had different means according to Duncan’s multiple comparisons with a global significance of 5%; ^(1), (2) and (3)^ show different means according to the C and Dennet multiple comparisons with a global significance of 5%; *descriptive level from ANOVA.

## DISCUSSION

In the present unprecedented study, we evaluated and compared two aspects that are relevant for clinicians who follow up with children and adolescents with musculoskeletal pain: pain perception and pain coping mechanisms.

Pain perception is the process by which an organism interprets and organizes sensations in order to give it meaning. Pain perception is not directly related to an active disease (the presence of arthritis or painful spots the body), but rather to a number of factors, such as the individual’s previous experience, social context, emotional state, gender, race, age and culture.^17^

The pain perception evaluation, which was performed through the presentation of simulated situations in the form of vignettes, showed that the JFM group had the highest scores (the highest pain perception) for the three situations presented, when compared to the other groups. The perception was greater for both the patients and their parents. The correlation between parents and children’s pain perception was low in all three groups. As such, the patients’ point of view should be considered.

In the comparison of the three vignettes within the same group, we can see how previous lived situations can influence pain perception. Evaluating each group separately, we observed that, in the JFM group, the highest scores for pain perception were given in reference to the social deprivation simulation, a situation that these patients know well due to the limitations imposed by their pain.

With the vignettes we were able to observe two aspects:

1. the pain perception scores in the JFM group were much higher than in the other groups in all of the simulated situations;
2. pain perception, when analyzing each individual group, is strongly influenced by the personal experience of each person.

In the JIA group, the highest score obtained from the vignettes was for the social isolation simulation, followed by physical trauma and then, receiving a shot. All of the children and adolescents who made up the JIA group received shots regularly to inject medication, controlling disease activity, and for laboratorial exams. Because injecting medication may be a positive experience for these patients, it could have influenced the lower pain perception score. Similarly, the situations of isolation and physical restraint that are caused by JIA in certain moments may have influenced the higher score for the social deprivation vignette.

The control group had the highest scores for pain perception from the shot simulation, followed by social deprivation and then, bicycle trauma. Healthy children and adolescents are not constantly subjected to experiences such as injectable medication. Many of these children were only given injections during their routine vaccination. We emphasize that this finding is unprecedented, since we did not find other articles aimed at measuring the perception of pain in hypothetical situations. The use of the vignettes designed in our study is still not well established in daily clinical practice. We believe that with the implementation of this instrument in the everyday routine of our outpatient clinic, we can obtain useful information to better design individual treatment plans.

The study by Varni et al.^15^ evaluated the relationship between previous pain experiences and the use of coping techniques in pediatric patients with rheumatic disease who had with musculoskeletal pain. According to this author, coping strategies are key in the relationship between pain perception and a well functioning child. The more pain coping tools or mechanisms an individual possesses, the less painful the perception of their pain will be, and the better their chances are of remaining functional. Several researchers have suggested that, for musculoskeletal pain, the strategies the child adopts to deal with pain play an important role in determining the child’s quality of life.^18^ ^,^ ^19^

JIA, despite being an inflammatory disease, can structurally comprise the joints, tendons and muscles, making remission difficult. Illowite et al.^20^ observed that joint inflammation only accounts for only 10% of the variation in pain scores. Furthermore, they found no significant relationship among disease subtypes or number of joints affected from the pain scores. In a study conducted by Hagglund et al.^21^ no association between disease severity and pain scores in children with JIA was observed. In our JIA group, we did not find a statistical difference for the pain score among the active and inactive subgroups of the disease, which is a reason why we considered it as a single and homogeneous group. In many cases, patients with JIA present little or no pain,^6^ especially when treatment is performed early in the diagnosis and is performed adequately, based on current protocols.^22^ Pain in the JIA patient group was reported to be transient - present for a few minutes upon waking up and at the start of a physical activity.

It is commonly known that pain is a preponderant factor in patients with musculoskeletal pain that is associated with amplification, as in the case of FMJ. In our study, the JFM group had the highest pain scores when compared to the other groups. More than 50% of the members of the JFM group reported intense pain, with a pain score higher than 8 in the week prior to the interview. On the other hand, for the JIA group, this figure was lower (22%). This difference in the proportion of patients with intense pain between the groups confirmed our impressions formed in day-to-day practice: patients with JFM complain more of intense and persistent pain that interferes in daily activities in comparison to patients with JIA.

Our data show that the parents of patients with JFM are able to measure the pain reported by their children better than the parents of patients with JIA. Pouchot et al.,^23^ in a study evaluating the perception of relatives and physicians of 7,700 patients with rheumatoid arthritis, observed that family members tend to overestimate the patient’s pain, while the attending physicians tend to underestimate the pain intensity.

Sawyer et al.^24^ observed that the coping mechanisms most used by children with JIA are cognitive self-instruction and problem-solving. In our study, patients with JIA and JMF used cognitive self-instruction and distraction techniques more efficiently than the control group. The AIJ group used techniques such as problem-solving, seeking social support and catastrophizing/helplessness as pain coping mechanisms.

Thastum et al.^25^ studied the relationship between pain coping strategies and previous pain experiences in children with JIA. These authors concluded that the higher the pain score, the greater the chance the child had to use mechanisms such as catastrophizing/helplessness to face the pain. In our study, this data cannot be confirmed. Patients with JIA used a greater number of coping techniques, perhaps because this group had been exposed to painful situations for a longer period of time or because they were given advice about some coping techniques during their treatment.

One of the limitations of our study is the fact that we evaluate subjective and personal data - pain - for which the gold standard is through self-reporting. Throughout the study we were careful to avoid external interference. All interviews were conducted by the same researcher (MMF), and the order in which the questionnaires were applied and vignettes were presented stayed the same for all the participants. The entire study was performed in the same physical space (the pediatric rheumatology clinic).

Patients with JIA and JFM behave differently in relation to pain perception and the development of pain coping techniques. In our study, patients with JIA had experienced pain longer than patients with JRM, since the pain started earlier in the JIA group. This group developed a greater number of mechanisms for coping with pain. The more time exposed to pain could have contributed to the development of more coping skills.

Pain originates from many factors and is influenced by several aspects while it lasts. We must evaluate the patient in pain from different perspectives. We have shown in our study that it is not enough to only measure pain intensity, we also need to deeply evaluate other aspects such as pain perception and pain coping mechanisms. Once each patient’s profile has been identified, it is the responsibility of all of the members of the multidisciplinary team to design an individualized therapeutic plan capable of relieving pain in its multiple form, with an emphasis on the physical, emotional, social and cognitive aspects.
